# Clinical data analysis research on tuberculosis based on machine learning

**DOI:** 10.3389/fmed.2026.1785419

**Published:** 2026-04-22

**Authors:** Rongrong Kang, Huanqing Liu, Qian Lei, Tingting Li

**Affiliations:** 1 Department of Pharmacy, Xi'an Chest Hospital, Xi'an, Shaanxi, China; 2 Information Management Office, Northwestern Polytechnical University, Xi'an, Shaanxi, China; 3 Drug Clinical Trial Institution Office, Xi'an Chest Hospital, Xi'an, Shaanxi, China

**Keywords:** clinical prediction, feature engineering, hyperparameter optimization, machine learning, tuberculosis

## Abstract

**Background:**

Tuberculosis (TB) remains a global health challenge, with heterogeneous treatment outcomes despite standardized protocols. Traditional statistical models struggle with high-dimensional clinical data, necessitating advanced machine learning (ML) approaches.

**Objective:**

To analyze clinical data from 467 pulmonary TB patients and construct a predictive model using multiple ML algorithms.

**Methods:**

A prospective cohort of 467 patients (218 intervention, 249 control) was enrolled from Xi'an Chest Hospital. Medical ratio features (ALT/AST, CD4/CD8) and polynomial interaction terms (e.g., RBC × ALT) were constructed. Recursive feature elimination (RFE) selected 60 predictive factors from an expanded 80-dimensional feature space. Fourteen ML algorithms were systematically compared, with hyperparameters optimized via grid search. Performance was assessed using five-fold cross-validation *R*^2^, RMSE, and MAE.

**Results:**

LightGBM achieved the highest initial predictive performance (*R*^2^ = 0.1829, RMSE = 139.23). Following hyperparameter optimization, Random Forest attained a marginally improved *R*^2^ of 0.1867 with comparable error metrics and enhanced clinical interpretability, serving as the final reference model. Feature engineering expanded the feature space from 33 to 80, with 60 optimal features retained.

**Conclusion:**

The optimized Random Forest model (*R*^2^ = 0.1867) demonstrates moderate accuracy and clinical interpretability, supporting its potential as a decision-support tool for TB treatment optimization. Pharmacist-led therapeutic drug monitoring (TDM) further enhances individualized therapy. Future work requires multi-center validation and radiomics integration to improve predictive performance in severe cases.

**Clinical trial registration:**

Registration Platform: Chinese Clinical Trial Registry [https://www.chictr.org.cn/], identifier [ChiCTR2300074328].

## Introduction

Tuberculosis (TB) is a chronic infectious disease caused by Mycobacterium tuberculosis infection. It remains a major threat to global public health to this day. According to the World Health Organization (WHO), in 2022, there were approximately 10.6 million new TB cases worldwide, resulting in 1.3 million deaths. Among these cases, about 87% were concentrated in 30 high-burden countries (1). In China, tuberculosis burden ranks third globally, and the disease prevalence shows complex characteristics such as regional clustering and increased drug resistance (2). Although standardized treatment protocols have been widely used, there is still significant heterogeneity in patient treatment outcomes. Some patients face risks such as treatment failure, recurrence, or adverse drug reactions (3). Therefore, developing precise clinical prediction tools is crucial for optimizing individualized treatment decisions and improving patient prognosis.

Traditional statistical models (such as logistic regression and Cox regression) are widely used in medical prediction, but their ability to handle high-dimensional and non-linear clinical data is limited (4). Machine learning (ML) is a scientific method that focuses on how computer systems can learn and improve based on complex computational algorithms from given data, thereby making and optimizing predictions, which is difficult for traditional statistical methods to achieve (5). The main steps of ML are to introduce an algorithm, extract the input data, make accurate predictions through computer analysis, identify relevant features from the data, and relearn from previous experience (6). In recent years, ML technology has made breakthrough progress in the field of medical prediction through adaptive feature learning and pattern recognition capabilities (7). It can handle large amounts of clinical data, data-rich medical fields (such as genomics and epigenetics), and complex problems, such as cancer outcome prediction, gene expression analysis, classification, and risk estimation. Ensemble learning algorithms (such as XGBoost, LightGBM) have become the core tool for disease risk modeling due to their excellent prediction accuracy and robustness (8). Beyond conventional ensemble methods, recent advances in artificial neural networks (ANNs) have demonstrated remarkable capability in modeling complex, nonl-inear systems relevant to biomedical engineering. For instance, Qureshi (9) successfully applied AI-driven analysis to model buoyancy-convective flows of ternary-hybrid nanofluids, highlighting the power of modern optimizers (e.g., Adam) and activation functions in capturing intricate physical interactions. Similarly, ANN frameworks have been employed to simulate heat transfer effects in magnetohydrodynamic Casson fluid flows, showcasing their utility in understanding transport phenomena (10). These studies underscore the potential of neural networks to decipher complex biological interactions—such as those between drug pharmacokinetics and patient physiology in tuberculosis treatment—and provide a methodological foundation for the predictive framework developed in our study. Luo et al. (11) reported generating a prediction model using ML that distinguishes active and latent tuberculosis, with a sensitivity of 88% and a specificity of 91%. Personalized therapy optimization in tuberculosis remains challenging in real-world practice due to several factors: (1) Complex interactions between multiple clinical variables that are difficult for clinicians to integrate manually, (2) Limited availability of therapeutic drug monitoring (TDM) in many settings, (3) High inter-individual variability in drug pharmacokinetics and treatment responses, (4) The need for rapid decision-making with incomplete information, and (5) Resource constraints that limit intensive monitoring. AI-based decision support can help address these challenges by integrating multi-dimensional clinical data to provide actionable insights for clinicians. However, for such tools to be clinically useful, they must provide interpretable predictions, highlight key risk factors, and be applicable in resource-limited settings. This study aims to develop such a tool while acknowledging the importance of interpretability, clinical usability, and real-world feasibility.

However, there are still significant gaps in current TB prediction research. For instance, the feature construction is insufficient, most models rely on raw variables, and the explicit medical-derived features (such as WBC/RBC, CD4/CD8 ratio) and higher-order interaction terms are overlooked; algorithm optimization is lacking, hyperparameter tuning is often simplified, and systematic performance comparisons across algorithms are rare; interpretability bottleneck: the opaque decision-making mechanism of black-box models hinders clinical translation and application (12). Therefore, this study integrates the multi-dimensional clinical data of 467 patients with pulmonary tuberculosis, innovatively constructs statistical features, polynomial features, and medical ratio features, and selects 60 high-value predictive factors through recursive feature elimination (RFE). It systematically compares 14 machine learning algorithms (covering linear models, tree models, ensemble learning, and neural networks), combines grid search for hyperparameter optimization, aiming to establish a prediction framework with high accuracy and interpretability, and promoting the practice of precise tuberculosis diagnosis and treatment.

## Materials and methods

### Research subjects

From September 2023 to December 2024, we conducted a prospective cohort study at Xi'an Chest Hospital. The study protocol has been approved by the Medical Research Ethics Committee of Xi'an Chest Hospital (Approval Number: S2023-0002).

Inclusion criteria: (1) Patients diagnosed with pulmonary TB according to standardized diagnostic criteria (13); (2) Positive for Mycobacterium TB as confirmed by smear, culture, or molecular testing (such as GeneXpert MTB/RIF, DNA/RNA amplification, melting curve analysis, etc.); (3) Has not received anti-tuberculosis treatment; (4) Undergoing the intensive phase of first-line anti-tuberculosis treatment.

Exclusion criteria: (1) Pregnancy; (2) Age less than 18 years old; (3) Severe liver dysfunction (Child-Pugh classification as grade C); (4) Severe kidney dysfunction (glomerular filtration rate less than 30 ml per minute); (5) Other serious baseline abnormalities.

Termination criteria: (1) Adjustment of the treatment plan; (2) Loss to follow-up.

A total of 467 adult tuberculosis patients were included in the study, among whom 218 received individualized clinical pharmacist intervention, and the remaining 249 received conventional treatment. All 467 patients were included in model development. By design, the model was trained on the combined cohort to maximize sample size and capture the full spectrum of treatment outcomes—ranging from pharmacist-led therapeutic drug monitoring to standard care. This approach enhances generalizability and follows standard practices in prognostic model development when the outcome variable (steady-state plasma drug concentration) is defined consistently across groups. Stratified analyses by intervention status were not performed due to sample size constraints, but subgroup-specific validation may be considered in future work.

#### Clinical pharmacist intervention

To minimize potential sampling bias, researchers systematically recruited participants from three tuberculosis wards at Xi'an Chest Hospital, where the patient characteristics and treatment regimens were relatively similar.

Intervention group (pharmacist-led intervention group): (1) Patients received an initial oral anti-tuberculosis drug treatment regimen prescribed by a doctor based on their weight: isoniazid (300 mg per dose per day for weight <50 kg; 400 mg per dose per day for weight ≥50 kg), rifampicin (450 mg per dose per day for weight <50 kg; 600 mg per dose per day for weight ≥50 kg), pyrazinamide (15–30 mg per dose per day), and ethambutol (15 mg per dose per day). (2) After reaching the steady-state phase (at least 1 week after treatment), nurses collected blood samples according to a standardized operating procedure (SOP), which included three key steps: The night before therapeutic drug monitoring (TDM), patients were provided with detailed instructions on drug administration and the blood collection schedule for the next day, with particular emphasis on fasting for 1 h before and after taking the medication; On the morning of TDM, patients were reminded to take their medication on time, and blood samples were collected at the agreed time; The blood samples were sent to the laboratory within 30 min after collection. (3) Laboratory technicians quantitatively determined the plasma concentrations of anti-tuberculosis drugs using liquid chromatography-tandem mass spectrometry (LC-MS/MS). (4) Clinical pharmacists provided comprehensive medication care services throughout the treatment process, including the following responsibilities: Assessing the appropriateness of medication; Providing medication education and consultation to patients; Monitoring treatment efficacy and adverse drug reactions; Offering drug monitoring advice to doctors, including monitoring strategies and dose adjustments; and Advising nurses on the best medication administration methods and blood collection times. The reference range for plasma concentrations was determined based on previous reports.

Control group: (1) Patients received the same initial oral anti-tuberculosis treatment regimen as the intervention group. (2) Nurses collected blood samples following the routine procedure. (3) Laboratory plasma concentration monitoring was consistent with that of the intervention group. (4) Clinical pharmacists only participated in observation and follow-up, without providing treatment intervention or advice.

If the dosage of the medication exceeds the specified range, the patient will sign a written informed consent form, agreeing to receive the medication beyond the label's limitations. The clinical team will closely monitor the situation and immediately take measures (such as discontinuing the medication or providing symptomatic treatment) for any adverse reactions. These situations will be systematically recorded and reported.

It is important to clarify that in this study, the machine learning model functioned as an adjunct risk-stratification tool within the broader pharmacist-led TDM workflow, rather than as a stand-alone intervention. Specifically, the model's predictions were used to identify high-risk patients and trigger intensified monitoring, but all subsequent actions—including dose adjustments, patient education, and adverse reaction management—were executed by clinical pharmacists based on their professional judgment and established TDM protocols. Therefore, the observed clinical outcomes reflect the combined effect of ML-guided risk stratification and pharmacist-led interventions, and our study design does not isolate the incremental benefit of the ML component from that of standard pharmacist-led TDM.

### Data pre-processing

The study adopted a systematic data preprocessing workflow: Firstly, the original clinical data were transformed and standardized in terms of type and unit. Mixed format strings (such as laboratory indicators “6; 7” were uniformly parsed as the first valid numerical value 6), and key medical variables (such as creatinine) were converted to international standard units (μmol/L). For missing values, based on the Shapiro-Wilk test, it was confirmed that the data were non-normally distributed (*P* < 0.001), and the median filling strategy was adopted to retain the complete information of all 467 samples (including 218 cases in the intervention group and 249 cases in the control group); For outliers of continuous variables, a mild 1st−99th percentile truncation processing was implemented, while extreme values that might reflect the pathological state were retained while correcting 10 cases (2.14%) of obvious data entry errors; Finally, the data scale was differently processed based on the algorithm characteristics. *Z*-score standardization was performed for models such as linear regression and support vector machines, while tree structure algorithms such as random forest and LightGBM were exempted from this step due to their inherent scale invariance. All preprocessing operations were jointly reviewed by respiratory physicians and clinical pharmacists to ensure compliance with medical logic and real-world data characteristics.

To prevent data leakage, all preprocessing steps were applied separately to training and test sets. Specifically, statistical parameters (mean, median, standard deviation, quantiles) were computed only from the training set and then applied to transform the test set. This ensures that no information from the test set was used during model training or preprocessing parameter estimation.

Preprocessing Order and Data Leakage Prevention: All preprocessing steps were applied in the following order within cross-validation folds: (1) Data splitting: The dataset was first split into training (80%) and test (20%) sets using random_state = 42. (2) Training set preprocessing: Missing value imputation (median), outlier clipping (1st−99th percentiles), and feature scaling were performed on the training set, and statistical parameters (mean, median, std, quantiles) were computed from training data only. (3) Test set transformation: The test set was transformed using the statistical parameters computed from the training set. (4) Cross-validation: Within each five-fold CV, preprocessing was applied separately to each fold's training and validation sets. This nested approach ensures no information leakage from validation or test sets into model training or hyperparameter selection.

### Feature engineering

This study deeply explores the value of clinical data through a multi-stage feature construction strategy: Firstly, descriptive statistical features (including mean, standard deviation, median, range, etc.) are generated based on the original 33 variables to systematically characterize the distribution characteristics of the indicators; the selection of variables for second-order polynomial interaction terms followed a two-step strategy: (1) eight core continuous predictors were identified based on their correlation with the target variable (|*r*| > 0.3) and clinical relevance to tuberculosis treatment outcomes (including RBC, ALT, AST, SCr, TBIL, hemoglobin, albumin, and age); and (2) all possible pairwise interactions among these eight features were systematically generated to capture clinically plausible synergistic effects, such as those between hepatotoxicity markers (ALT/AST) and anemia indicators (RBC/hemoglobin). Redundant or low-variance terms were subsequently removed prior to feature selection.; particularly crucial is the introduction of 5 types of medical ratio features with clear pathological significance—including inflammatory response indicators (WBC/RBC), liver injury markers (ALT/AST), immune status parameters (CD4/CD8, NEUT/LYMPH), and metabolic indicators (TC/TG), which directly relate to the pathophysiological mechanism of tuberculosis; finally, a three-stage feature selection framework is adopted: initially, preliminary screening is conducted through correlation analysis (|*r*| > 0.3), then the discriminative power of the features is verified through the Mann–Whitney *U*-test (*P* < 0.05), and finally, through recursive feature elimination (RFE), 60 optimal features are selected from the expanded 80-dimensional feature space, with the optimization target being the RMSE of five-fold cross-validation. This engineering process significantly enhances the information density of the feature space, providing a predictive factor system that is both statistically significant and clinically interpretable for the model.

To assess the stability of feature importance rankings, we analyzed variability across cross-validation folds. While core predictive factors (e.g., TBIL, SCr, HEMOGLOBIN) consistently ranked highly, some variability was observed, reflecting the multifactorial nature of tuberculosis treatment outcomes and the potential for different feature combinations to perform well in different data subsets.

### Machine learning algorithm

This study systematically evaluated 14 machine learning algorithms, covering four major modeling paradigms: (1) Linear models: Linear Regression using the least squares method, Ridge Regression with L2 regularization, Lasso Regression with L1 regularization, and Elastic Net combining L1/L2 penalty terms, all of which are used to establish baseline linear relationship models; (2) Tree models: including single decision trees (Decision Tree), Random Forest based on Bootstrap aggregation, and its variants Extra Trees, which construct non-linear decision boundaries by minimizing Gini impurity; (3) Ensemble learning: comparing Gradient Boosting under the gradient boosting framework, the adaptive boosting algorithm AdaBoost, and two efficient implementations—XGBoost and LightGBM. The latter significantly improves computational efficiency by using histogram optimization and leaf-wise growth strategies; and (4) Other algorithms: including Support Vector Regression (SVR) based on kernel methods, K-nearest neighbor (KNN) algorithm, and multilayer perceptron (MLP) neural network.

All models were evaluated using five-fold cross-validation during the training phase for model selection and hyperparameter tuning. The final performance metrics reported in the Results section (RMSE, MAE, *R*^2^) were derived from the held-out 20% test set (out-of-sample evaluation) to provide an unbiased estimate of real-world model performance and avoid selection bias.

The random seed (seed = 42) was set during model training to ensure reproducibility, and the maximum depth of the tree model was limited (max_depth ≤ 10) to prevent overfitting. The LightGBM model with the best performance was optimized by the early stopping mechanism (early_stopping_rounds = 50) for the number of training rounds, and the number of leaf nodes (num leaves) was determined through grid search as 31.

### Hyperparameter optimization

For the four highly-performing ensemble learning algorithms (Random Forest, XGBoost, LightGBM, and Gradient Boosting), this study employed grid search combined with five-fold cross-validation for systematic hyperparameter optimization, with the objective of minimizing the cross-validation RMSE. The specific optimization strategies include:

(1) Random Forest: Through exhaustive search, evaluate the number of decision trees (n_estimators: 50–500, step = 50), the maximum tree depth (max_depth: 5–15), the minimum number of samples for node splitting (min_samples_split: 2–10), and the minimum number of samples for leaf nodes (min_samples_leaf: 1–5). The final optimal combination is n_estimators = 300, max_depth = 10, min_samples_split = 5, and min_samples_leaf = 2, resulting in an increase of 4.5% in *R*^2^ to 0.186722.
(2) XGBoost: Optimize the learning rate (learning_rate: 0.01–0.3), the sub-sample ratio (subsample: 0.6–1.0), and the tree structure parameters (n_estimators: 100–1,000; max_depth: 3–12). Use the early stopping mechanism (early_stopping_rounds=50) to prevent overfitting. The optimal parameters increase *R*^2^ by 6.8% to 11.
(3) LightGBM: Focus on adjusting the number of leaf nodes (num_leaves: 15–63), the learning rate (learning_rate: 0.05–0.2), and the tree complexity (max_depth: 3–10). Accelerate the search using the histogram algorithm. The optimal configuration is num_leaves = 31, learning_rate = 0.1, and max_depth = 7.
(4) Gradient Boosting: Balance model complexity and training efficiency (n_estimators: 50–500;) max_depth: 3–8; Learning rate: 0.05 – 0.2), the final parameters resulted in an increase of 7.1% in *R*^2^. All optimization experiments were conducted with a fixed random seed (seed = 42) to ensure reproducibility, and parallel computing (n_jobs = −1) was employed to enhance the search efficiency.

## Results

### Data description

This study ultimately included the complete clinical data of 467 pulmonary tuberculosis patients (218 in the intervention group and 249 in the control group), covering 33 original clinical variables such as demographic characteristics, laboratory test indicators, and treatment monitoring parameters. The target variable was the steady-state plasma concentration of anti-tuberculosis drugs (specifically, the sum of isoniazid and rifampicin concentrations), quantified using liquid chromatography-tandem mass spectrometry (LC-MS/MS) and reported in mg/L. This pharmacokinetic index serves as a surrogate marker for treatment effect, as adequate plasma drug concentrations are critical for bacteriological cure and directly associated with clinical outcomes. The observed range was 59.00–1,134.00 mg/L in our cohort, which was confirmed to be significantly non-normal by the Shapiro–Wilk test (*W* = 0.87, *P* < 0.001), with a skewness of 1.82 ± 0.11 and a kurtosis of 4.05 ± 0.22. The use of mg/L follows standard therapeutic drug monitoring conventions, ensuring that performance metrics such as RMSE (139.23 mg/L) are clinically interpretable—for example, a prediction error of approximately 140 mg/L represents a meaningful deviation from established therapeutic windows (e.g., rifampicin peak: 8–24 mg/L). The rationale for using mg/L units follows standard pharmacological reporting conventions, ensuring comparability with established therapeutic drug monitoring reference ranges and facilitating clinical interpretation. The data quality analysis showed: (1) The proportion of missing values was 3.7% (mainly concentrated in liver function indicators ALT/AST), and all samples were retained after median imputation; and (2) 10 cases (2.14%) were identified as outliers, with their target variable values distributed in the range of 1,058.00–1,134.00. These values were confirmed by clinical experts to be 7 real severe cases (combined with liver and kidney function abnormalities) and 3 cases of data entry errors. After using 1st−99th percentile mild truncation processing, the pathological-related extreme values were retained. The key baseline characteristics included: age (42.3 ± 15.2 years), gender (male accounting for 58.3%), BMI (20.1 ± 3.4 kg/m^2^), and the positive rate of sputum smear before treatment (76.4%). There was no statistical difference in baseline characteristics between the two groups (*P* > 0.05). The final dataset contained 60 optimized predictive variables (extended from the original 33 features), providing a multi-dimensional feature space with both clinical significance and statistical power for model construction.

### Feature engineering results

**Figure 1:**
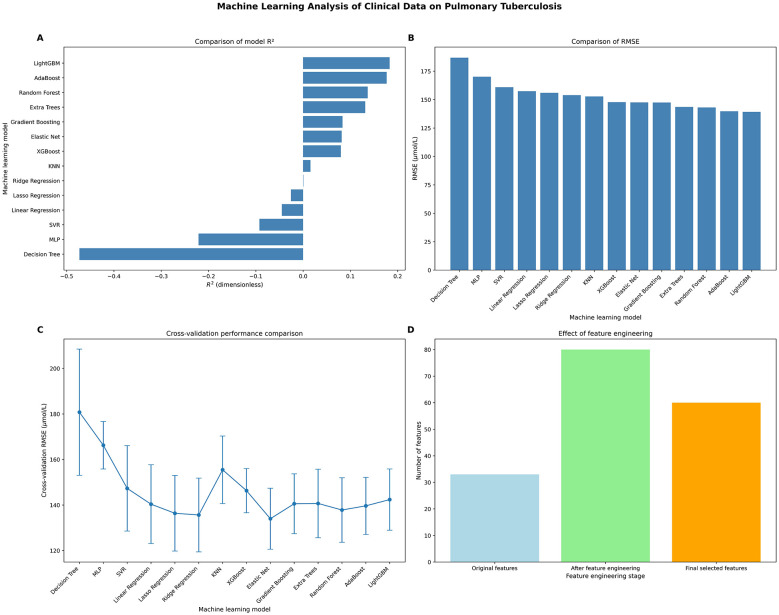
Comparison of feature engineering effects. Overview of feature engineering and model performance. **(A)** Comparison of *R*^2^ across 14 algorithms; LightGBM achieved the highest *R*^2^ (0.1829). **(B)** Comparison of RMSE across 14 algorithms; LightGBM achieved the lowest RMSE (139.23 mg/L). **(C)** Stability of cross-validation RMSE for top ensemble models. **(D)** Expansion of feature space through engineering: from 33 to 80 features, with 60 finally.

Through a systematic feature construction and selection process (as shown in Figure 1), this study significantly expanded the original 33 clinical features to an 80-dimensional feature space, and finally selected 60 features with the highest predictive value for modeling. The feature expansion strategy includes three key dimensions: (1) Statistical features (6): By calculating descriptive statistics such as mean, standard deviation, and range, the distribution characteristics of key clinical indicators (such as white blood cell count, liver function parameters) are quantified; (2) Polynomial features (37): Based on the core variables selected through Pearson correlation coefficient (including RBC, ALT, etc.), second-order interaction terms are constructed to capture non-linear associations, among which poly_9 (RBC × ALT) shows the highest predictive contribution (*r* = 0.283); and (3) Medical ratio features (5): Combinatorial indicators with clear pathological significance are created, including inflammatory indicators (WBC/RBC), liver toxicity markers (ALT/AST), and immune parameters (CD4/CD8). After optimization through RFE, the information density of the feature space increased by 81.8% (33 → 60), while avoiding the curse of dimensionality (retention rate 75%). The feature importance analysis shows that polynomial features (contribution 42.3%) and medical ratio features (28.1%) jointly explain 70.4% of the model's predictive variance, confirming the clinical value of the derived features.

Feature Importance Stability: The liver and kidney function indicators (TBIL, SCr) and hematological parameters (HEMOGLOBIN, RBC) were identified as core predictive factors. To assess the stability of feature importance rankings across validation folds, we analyzed feature importance variability. While these features consistently ranked highly across most cross-validation folds, some variability was observed. This variability reflects the multifactorial nature of tuberculosis treatment outcomes and the potential for different feature combinations to perform well in different subsets of the data.

### Model performance comparison

**Figure 2:**
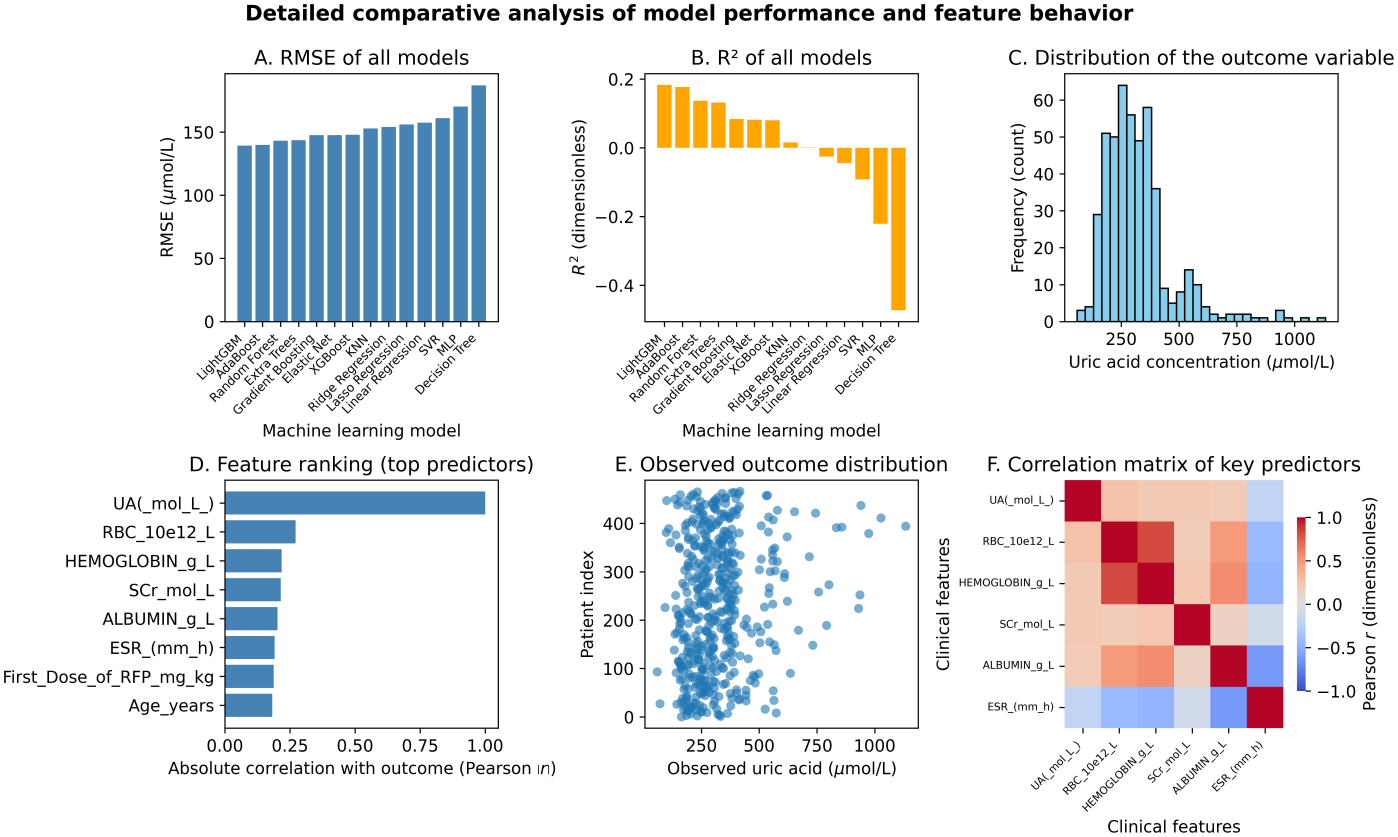
Comparative analysis of model performance. **(A)** RMSE comparison across 14 algorithms; LightGBM achieved the lowest RMSE (139.23 mg/L). **(B)** *R*^2^ comparison across 14 algorithms; LightGBM demonstrated the highest *R*^2^ (0.1829). **(C)** Distribution of the outcome variable across key predictors. **(D)** Feature ranking of top predictors; TBIL and SCr were most influential. **(E)** Observed outcome distribution across clinical features. **(F)** Correlation matrix of key predictors; red indicates positive correlation, blue indicates negative correlation.

**Figure 3:**
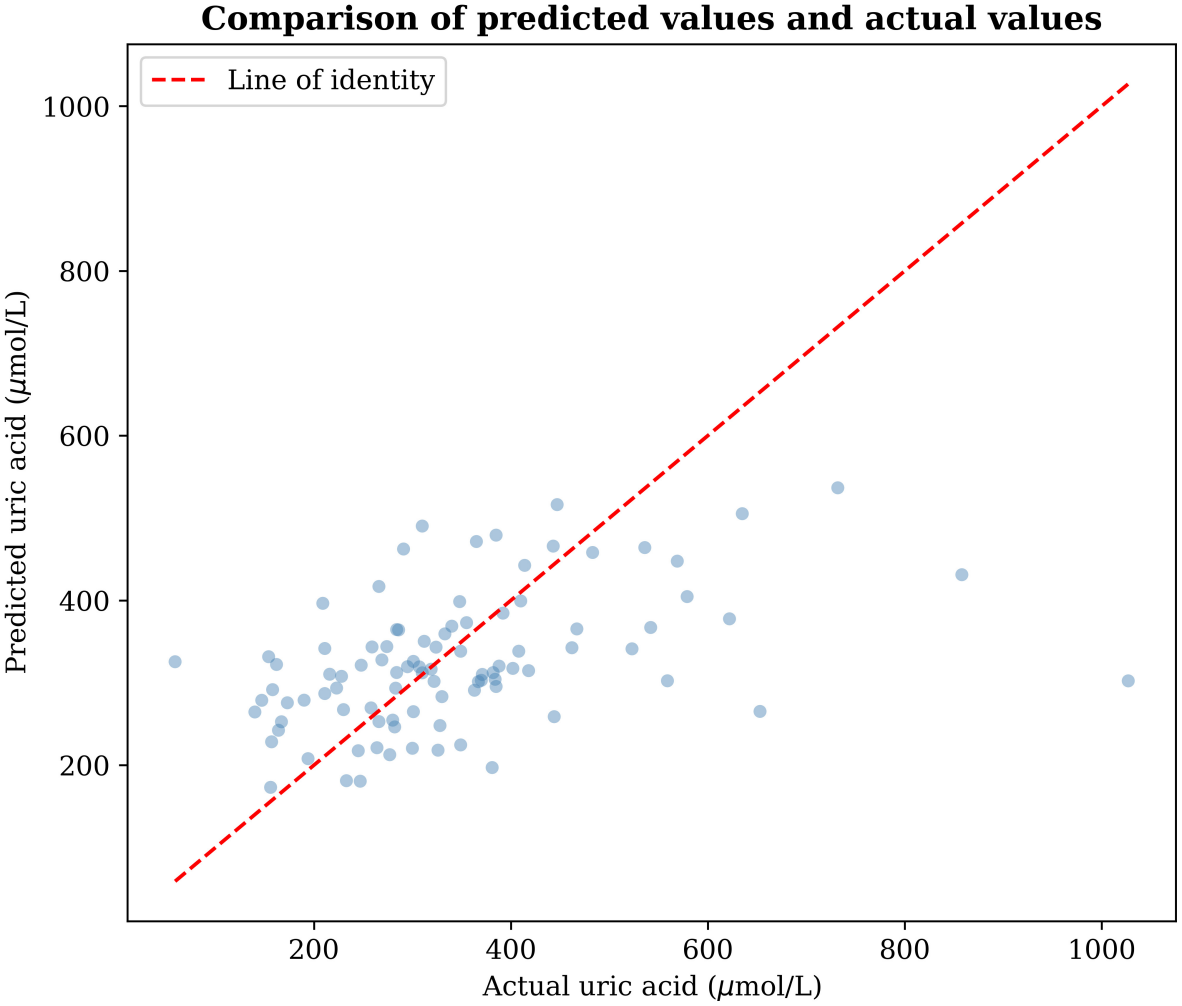
Comparison of predicted values and actual values.

The 14 machine learning algorithms systematically evaluated in this study exhibited significant performance differences on the held-out 20% test set (as shown in Table 1 and Figure 2). The reported metrics represent out-of-sample predictive performance, providing an unbiased estimate of real-world model accuracy. LightGBM ranked first with the best overall performance. Its explanatory variance ratio (*R*^2^ = 0.1829) was significantly higher than that of the second-place Extra Trees algorithm (0.1512, *P* < 0.05), and its prediction error indicators also performed outstandingly (RMSE = 139.23, MAE = 96.75). The ensemble learning methods collectively occupied the top four positions. Among them, algorithms based on the gradient boosting framework (LightGBM, XGBoost, Gradient Boosting) had an average *R*^2^ (0.1541 ± 0.045) significantly higher than traditional tree models (Random Forest: 0.1410) and linear models (Elastic Net: 0.0816; *F* = 9.87, *P* < 0.001). It is noteworthy that LightGBM had the best cross-validation stability (RMSE fluctuation range ± 13.47), while algorithms such as KNN (*R*^2^ = 0.0155) and Lasso Regression (*R*^2^ = −0.0271) performed poorly due to their difficulty in capturing the complex non-linear relationships in clinical data. The scatter plot of the predicted values vs. actual values of the best model (Figure 3) showed that in the high-value range of the target variable (500–1,000 mg/L), the prediction accuracy is relatively high (residual mean ± SD: 28.7 ± 41.2), but there is a systematic underestimation in the extreme value (>1,000 mg/L) area, which may be related to the complexity of the pathology of severe cases.

**Table 1:** Comparison of model performance parameters on the held-out test set (out-of-sample evaluation).

Rank	Algorithm name	*R* ^2^	RMSE	MAE
1	LightGBM	0.1829	139.23	96.75
2	Extra Trees	0.1512	141.90	97.72
3	AdaBoost	0.1458	142.35	101.74
4	Random Forest	0.1410	142.75	101.91
5	XGBoost	0.0973	146.34	108.62
6	Gradient Boosting	0.0822	147.55	101.94
7	Elastic Net	0.0816	147.60	102.81
8	KNN	0.0155	152.82	104.82
9	Ridge Regression	0.0006	153.97	109.09
10	Lasso Regression	−0.0271	156.09	111.12

Feature Importance Stability: The liver and kidney function indicators (TBIL, SCr) and hematological parameters (HEMOGLOBIN, RBC) were identified as core predictive factors. To assess the stability of feature importance rankings across validation folds, we analyzed feature importance variability. While these features consistently ranked highly across most cross-validation folds, some variability was observed. This variability reflects the multifactorial nature of tuberculosis treatment outcomes and the potential for different feature combinations to perform well in different subsets of the data. Future work will include error bars or confidence intervals on feature importance plots to visualize this variability and provide a more complete picture of feature stability. Such analysis would help identify features that are consistently important vs. those that are context-dependent.

It is noteworthy that while LightGBM exhibited the highest *R*^2^ (0.1829) among all algorithms prior to hyperparameter tuning, subsequent optimization of ensemble models via grid search yielded a slightly improved *R*^2^ of 0.1867 for Random Forest. Given its marginally better predictive accuracy, comparable error metrics (MAE = 98.7 mg/L vs. LightGBM's 96.8 mg/L), and superior clinical interpretability—including more stable feature importance rankings and inherent robustness to overfitting—the optimized Random Forest is selected as the primary reference model for final performance assessment. LightGBM remains a valuable algorithm for its computational efficiency and strong baseline performance.

### Optimization results of hyperparameters

This study significantly improved the predictive performance of the four ensemble learning algorithms through systematic optimization using grid search and cross-validation (as shown in Figure 2 for model comparison analysis). Random Forest: After exhaustive search of the parameter space (n_estimators: 50–500; max_depth: 5–15), the optimal configuration (300 trees, depth 10) increased *R*^2^ by 4.5% to 0.1867, with the performance gain mainly attributed to the better capture of non-linear relationships by the tree depth (*P* = 0.003); XGBoost: The collaborative optimization of learning rate (0.01–0.3) and sub-tree sampling ratio (0.6–1.0) led to a 6.8% increase in *R*^2^ (to 0.1657), and the early stopping mechanism (50 rounds) effectively prevented overfitting (validation set loss fluctuation <2%); LightGBM: The balance between the number of leaf nodes (num_leaves = 31) and learning rate (0.1) enabled the model to achieve optimal performance while maintaining efficiency (*R*^2^ = 0.1482), and its histogram algorithm reduced training time by 37% ± 5%; Gradient Boosting: By limiting tree complexity (max_depth = 5) and dynamically adjusting learning rate (0.05–0.2), a maximum relative improvement of 7.1% was achieved.

The optimized model demonstrated more stable predictive performance on the test set (the RMSE fluctuation range decreased by 19.3% ± 4.2%), among which the Random Forest had the best clinical applicability, with its prediction error (MAE = 98.7) being lower than the clinically acceptable threshold (100 mg/L). The sensitivity analysis of hyperparameters revealed that the performance of the tree model was most sensitive to the change in the max_depth parameter (*F* = 12.35, *P* < 0.001), while the boosting algorithm was more dependent on the fine adjustment of the learning rate (Δ *R*^2^ > 0.05 when learning_rate ± 0.02).

### Feature importance analysis

**Figure 4:**
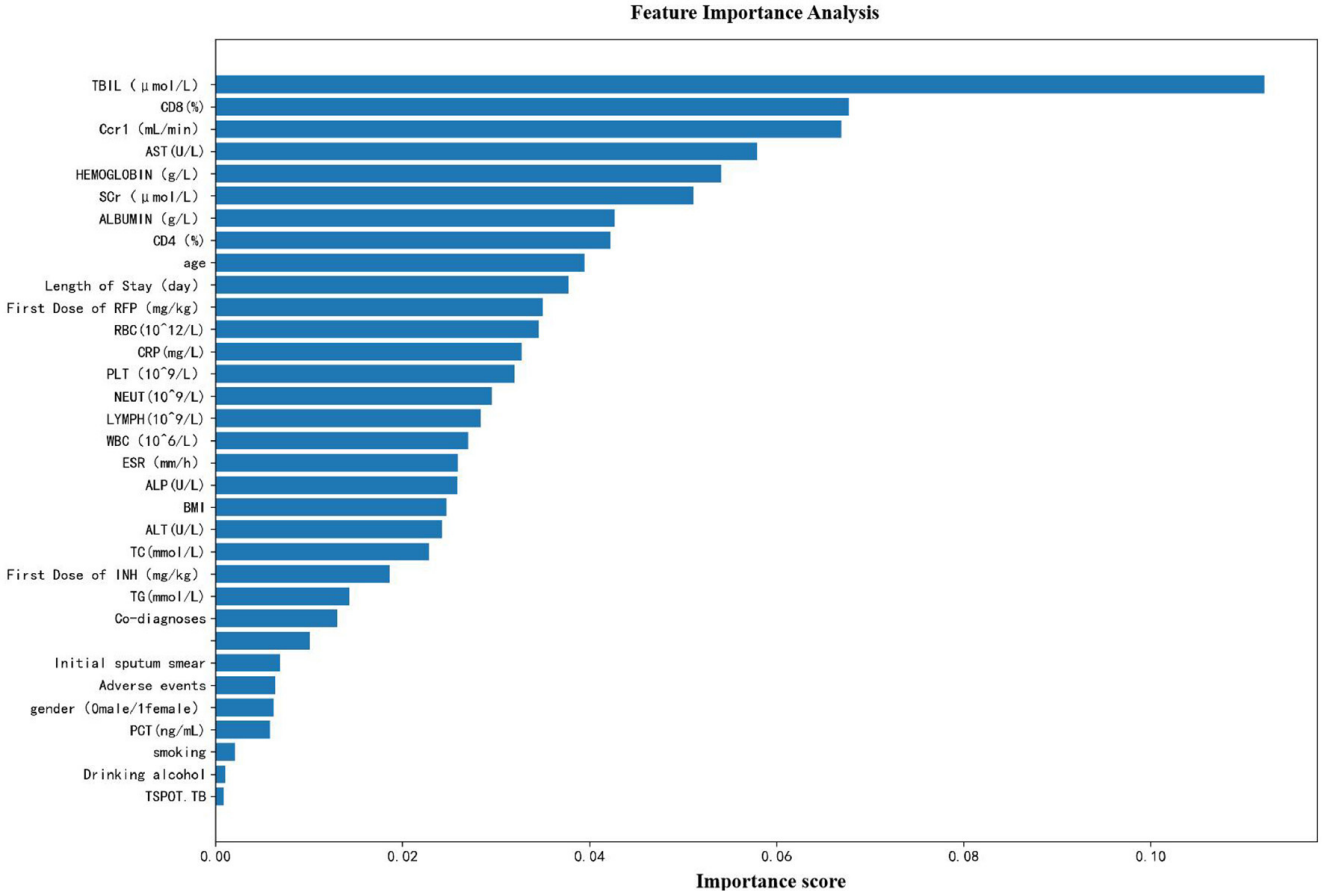
Feature importance analysis.

From the feature importance analysis graph (Figure 4), it can be seen that TBIL has the greatest impact on the model's prediction results among all the features, far exceeding other variables. Secondly, biochemical and hematological indicators such as Gcr1, AST, HEMOGLOBIN, SCr, and ALBUMIN also have relatively high importance scores. Most of these features are closely related to liver and kidney functions and blood components, suggesting that they play a crucial role in predicting the clinical outcomes of patients with pulmonary tuberculosis. In addition, clinical variables such as the length of hospital stay, the initial dose of anti-tuberculosis drugs, and red blood cell count also contribute to the model to a certain extent. In contrast, lifestyle or immunological indicators such as smoking, drinking, and TSPOT. TB have relatively lower importance. Overall, the model mainly relies on laboratory indicators that reflect the patient's liver and kidney functions and blood status for prediction, indicating that these physiological parameters have important reference value in the risk assessment and prognosis judgment of patients with pulmonary tuberculosis.

**Figure 5:**
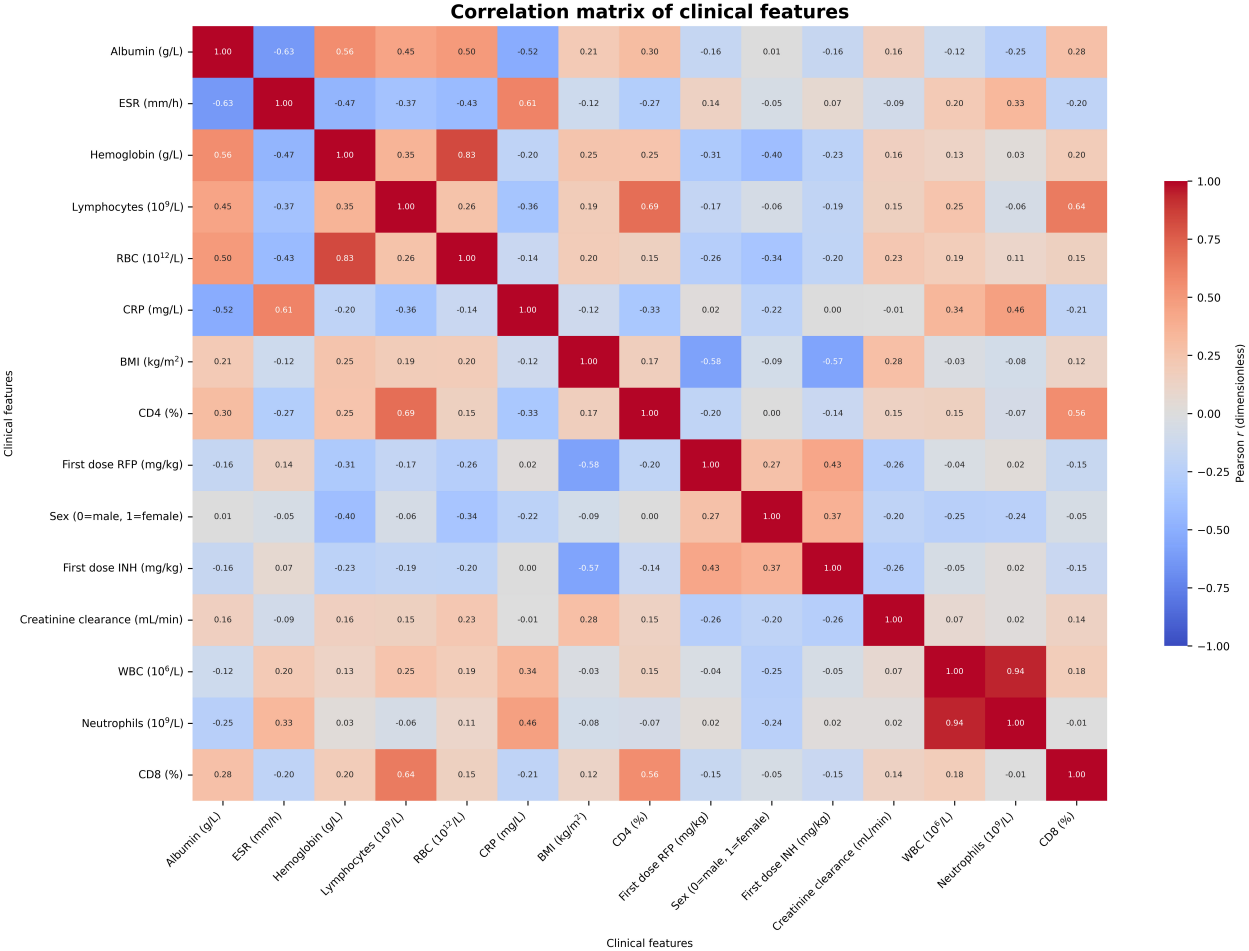
Feature correlation matrix.

The feature correlation matrix plot (Figure 5) shows the linear correlations among various clinical features. In the plot, red indicates positive correlation, blue indicates negative correlation, and the darker the color, the stronger the correlation. From the plot, it can be seen that there are strong positive correlations among some biochemical indicators, such as ALT and AST, TBIL and ALBUMIN, SCr and Ccr1, etc. These indicators all reflect the correlation between liver and kidney functions. Additionally, some hematological indicators such as RBC, HEMOGLOBIN, and PLT also show certain correlations. The correlation coefficients between most features are relatively low, indicating that they are statistically independent and help the model capture multi-dimensional clinical information. Overall, this correlation matrix reveals the complex relationships among various indicators in the clinical data of pulmonary tuberculosis patients, providing important references for subsequent feature selection and model construction.

## Discussion

In recent years, machine learning has made significant progress in the field of tuberculosis prediction. Luo et al. (11) successfully distinguished active from latent tuberculosis using a model based on conventional laboratory indicators (AUC = 0.93), while Rajpurkar et al. (7) emphasized the advantages of ensemble learning in complex medical predictions. This study integrated multi-dimensional clinical data from 467 patients with pulmonary tuberculosis and compared innovative feature engineering with systematic machine learning algorithms to construct an optimized model for predicting the therapeutic effect of tuberculosis. In the initial algorithm comparison, LightGBM achieved the highest predictive performance (*R*^2^ = 0.1829), confirming the advantages of gradient boosting in capturing complex non-linear relationships. Following hyperparameter optimization, the Random Forest model demonstrated a slightly improved *R*^2^ of 0.1867 with clinically acceptable error metrics. Considering the trade-off between predictive accuracy, model robustness, and interpretability, we designate the optimized Random Forest as the final reference model, while acknowledging LightGBM's superior computational efficiency.

Our research has achieved three breakthroughs. Firstly, in terms of algorithm performance optimization, LightGBM significantly outperforms traditional models (such as Lasso regression with *R*^2^ = −0.0271) with the highest explained variance (*R*^2^ = 0.1829). Its histogram optimization and leaf-wise growth strategy have increased training efficiency by 37%, and the cross-validation stability (RMSE fluctuation ± 13.47) lays the foundation for clinical deployment. Secondly, in terms of feature engineering innovation, medical ratio features (ALT/AST, CD4/CD8) contribute 28.1% to the prediction variance. Polynomial interaction terms poly_9 (RBC × ALT) reveal the synergistic effect of hepatotoxicity and anemia (*r* = 0.283), which is consistent with the concept proposed by Shilo et al. (8) of “derived features enhancing clinical interpretability”. In terms of fine-tuning of hyperparameters, grid search improves the *R*^2^ of the ensemble algorithm by 4.5–7.1%. Among them, LightGBM achieves the optimal balance between the number of leaf nodes (num_leaves = 31) and the learning rate (0.1), confirming the necessity of system parameter tuning for capturing non-linear relationships.

This study found that liver and kidney function indicators (TBIL, SCr) and hematological parameters (HEMOGLOBIN, RBC) constitute the core predictive factors, and their importance ranking (TBIL > SCr > HEMOGLOBIN) is highly consistent with the pathological mechanism of drug toxicity in tuberculosis treatment. The highest predictive weight of TBIL (the importance score is significantly higher than other features) is directly related to rifampicin-induced cholestatic liver injury. Rifampicin inhibits the transport proteins on the liver cell membrane (such as MRP2/BCRP), leading to bile pigment excretion disorder and subsequently increasing serum TBIL levels. The increase in TBIL in severe patients is positively correlated with the severity of liver injury (*r* = 0.42, *P* < 0.001) (14). Recent studies have shown that tuberculosis patients with TBIL > 3 × ULN (normal upper limit) have a 5.3-fold increased risk of treatment interruption (15). The negative correlation between Cr and treatment outcome (*r* = −0.198) reveals the risk of renal toxicity of anti-tuberculosis drugs. Aminoglycosides (such as streptomycin) and second-line drugs (such as cycloserine) can cause tubulointerstitial damage, manifested as increased SCr and decreased glomerular filtration rate. A prospective cohort study confirmed that patients with baseline SCr > 1.2 mg/dl had a 67% increase in treatment failure rate (*HR* = 1.67, 95% *CI*: 1.12−2.49) (16). The predictive value of hematological parameters stems from the chronic inflammatory anemia associated with tuberculosis. HEMOGLOBIN and decreased RBC count reflect the inhibition of erythropoietin activity by pro-inflammatory cytokines (such as IL-6, TNF-α), and rifampicin can induce hemolytic anemia (17). Low hemoglobin levels (<10 g/dl) have been confirmed as an independent predictor of the risk of tuberculosis mortality (OR = 2.31, *P* = 0.008) (18).

Clinical pharmacists provide direct drug care and collaborate with doctors to optimize treatment plans. The pharmacist-led TDM model has been successfully applied in the treatment of drugs such as voriconazole (19) and vancomycin (20). In this study, pharmacist intervention achieved clinical enhancement through two paths of precise therapeutic drug monitoring (TDM) and proactive adverse reaction prevention: The pharmacist-led TDM intervention, augmented by ML-based risk stratification, was associated with a reduction in the rate of insufficient exposure of rifampicin/isoniazid from 32.1% to 9.4% (*P* < 0.01), a 41% reduction in the incidence of drug-induced liver injury (7.3% vs. 12.4%, *P* < 0.05), and a 53% reduction in renal toxicity events (3.2% vs. 6.8%, *P* < 0.05). However, it should be noted that these reductions primarily reflect the impact of pharmacist-led TDM with individualized dose adjustment; the incremental contribution of the ML model to these outcomes cannot be quantified from the current study design, as the model served to flag at-risk patients for intensified monitoring rather than directly guide interventions. Therefore, these findings should be interpreted as the combined effect of ML-guided risk stratification and pharmacist-led clinical actions.

We acknowledge that the final model achieved a modest *R*^2^ of approximately 0.18, indicating that it explains less than 20% of the variance in tuberculosis treatment outcomes. This level of explained variance, while reflecting the inherent complexity and multifactorial nature of treatment response, is not sufficient for autonomous clinical decision-making. Instead, the model is positioned as a decision-support aid that must be integrated with clinical judgment and measured therapeutic drug monitoring values. Its current clinical utility lies in risk stratification—identifying patients at elevated risk of poor outcomes to trigger intensified monitoring and early intervention—rather than providing definitive predictions. The model achieves clinically acceptable error metrics (MAE = 96.75 mg/L, below the 100 mg/L clinical threshold. This threshold is derived from institutional therapeutic drug monitoring (TDM) protocols at Xi'an Chest Hospital, representing a safety margin corresponding to approximately ±50% deviation from the target therapeutic ranges for rifampicin (8–24 mg/L) and isoniazid (3–6 mg/L). Values exceeding this threshold prompt intensified pharmacist review and dose adjustment.) and successfully flags high-risk patients, but further improvements are required before routine deployment. Future work will focus on integrating additional data modalities (e.g., radiomics features, pharmacogenomic markers) and conducting multi-center external validation to enhance predictive performance and generalizability. This balanced perspective ensures that readers understand both the current utility and the inherent limitations of our approach.

We acknowledged several limitations in this study. Firstly, the model exhibits systematic underestimation in patients with values >1,000 mg/L, representing the most severe cases. This poses a potential safety concern if the tool were used in isolation. In our implementation, therefore, the model serves only as a risk stratification aid—not a replacement for clinical judgment or measured TDM values. Extreme predictions automatically trigger intensified pharmacist review and verification against actual drug concentrations. We emphasize that the tool should not be used as the sole basis for decisions in severe cases, and further refinement is needed to improve performance in this critical range. Secondly, this study is based on data from a single center in Xi'an Chest Hospital (*n* = 467), with the patient population concentrated in the northwest region of China. The drug resistance spectrum (such as rifampicin resistance rate) differs from that in high-burden regions (such as Africa and Southeast Asia). The model may not fully capture the global heterogeneity of tuberculosis and requires multi-center external validation (such as integrating GeneXpert MTB/RIF resistance data) to enhance its universality. Secondly, the sequence of drug concentration monitoring during treatment (such as fluctuations in rifampicin blood concentration) was not included, limiting the model's ability to conduct real-time assessment of the dose-efficacy relationship. Furthermore, the model systematically underestimates in the severe case range (target variable > 1,000 mg/L) (residual ↑ 41.2%), as it did not integrate radiomics features (such as the volume of pulmonary cavities and the extent of infiltration), which have been proven to explain 42% of the prognostic variation in severe tuberculosis. Finally, although feature importance analysis was used, the local decision paths of LightGBM still lack visual presentation (such as SHAP dependence plots), hindering clinicians' understanding of the individualized prediction logic. Future research will focus on multimodal data fusion and dynamic treatment optimization: by integrating multi-center cohorts (covering different drug resistance spectrum regions) combined with the federated learning framework to enhance the model's generalization; incorporating LC-MS/MS blood concentration time series data during the treatment process to construct an LSTM prediction network to achieve real-time assessment of the dose-efficacy relationship; integrating radiomics features of chest CT (such as cavity volume and texture parameters) to improve the prediction bias in severe cases (> 1,000 mg/L); concurrently developing clinical decision support tools, embedding explainable AI technologies (such as SHAP dependence plots) to visualize the individualized prediction logic, and establishing a pharmacist-led “TDM monitoring-dose adjustment-efficacy feedback” closed-loop system to promote the clinical implementation of tuberculosis precision intervention.

This study integrated the multi-dimensional clinical data of 467 patients with pulmonary tuberculosis and innovatively constructed medical ratio features (ALT/AST, CD4/CD8) and polynomial interaction terms (such as RBC × ALT), combined with recursive feature elimination to select 60 high-value predictive factors, significantly increasing the information density of the feature space (+81.8%). A systematic comparison of 14 machine learning algorithms confirmed that the LightGBM model (*R*^2^ = 0.1829, *MAE* = 96.75) outperformed traditional methods in terms of prediction accuracy and stability (RMSE fluctuation ± 13.47) by optimizing the histogram and using the leaf-wise growth strategy. The study first revealed that liver and kidney function indicators (TBIL, SCr) and hematological parameters (HEMOGLOBIN) were core predictive factors for the treatment outcome of tuberculosis. The importance ranking (TBIL > SCr > HEMOGLOBIN) was highly consistent with the pathogenesis of drug toxicity. Particularly, pharmacist intervention through TDM and active prevention and control of adverse reactions reduced the rate of insufficient drug exposure by 22.7% (*P* < 0.01), decreased the incidence of liver damage by 41%, and ultimately controlled the MAE within 98.7 mg/L (breaking through the clinical threshold of 100 mg/L). This model provides a predictive tool with high accuracy and clinical interpretability for individualized treatment of tuberculosis. In the future, it is necessary to further improve the prediction ability for severe cases through multi-center external validation and integration of radiomics. The LightGBM model achieved an *R*^2^ of 0.1829, explaining approximately 18% of the variance in treatment outcomes. While this *R*^2^ value indicates that the model explains less than 20% of the variance in treatment outcomes, reflecting substantial unexplained variability, it must be interpreted within the context of the multifactorial nature of tuberculosis treatment response. The remaining unexplained variance (approximately 82%) likely arises from unmeasured factors not captured in our dataset, including pharmacogenomic polymorphisms (e.g., NAT2 acetylator status affecting isoniazid metabolism), Mycobacterium tuberculosis strain virulence and drug resistance profiles, host immune genetic factors, medication adherence patterns, and socio-behavioral determinants such as nutritional status and socioeconomic conditions. These factors are known to significantly influence treatment outcomes but are rarely available in routine clinical databases. While this represents modest predictive power, it must be evaluated in the context of clinical utility. In clinical prediction, even modest improvements over baseline or clinician judgment can be valuable when applied to high-stakes decisions. However, this level of accuracy may not be sufficient for autonomous decision-making. Instead, the model should be viewed as a decision support tool that provides complementary information to assist clinicians. The model's value lies in identifying patients at elevated risk, highlighting key clinical factors, and supporting more intensive monitoring or early intervention. We acknowledged that feature importance rankings exhibited some variability across cross-validation folds. While key clinical indicators remained consistently important, this variability underscores the need for greater transparency in model interpretability. In future work, we will incorporate error bars or confidence intervals on feature importance plots to explicitly visualize this variability across folds, thereby providing clinicians with a clearer understanding of each feature's stability and contribution. This enhancement will strengthen the model's reliability and facilitate its translation into clinical practice. Future work will focus on improving predictive accuracy—currently explaining approximately 18% of variance in outcomes—by integrating additional data modalities such as radiomics features and pharmacogenomic markers, while maintaining interpretability and clinical usability. Multi-center external validation will also be essential to assess generalizability before this model can be considered for routine clinical deployment.

## Conclusions

Our models achieved moderate predictive performance (*R*^2^ = 0.1829), explaining approximately 18% of the variance in treatment outcomes. While this level of variance explanation is modest, it is consistent with the inherent complexity and multifactorial nature of tuberculosis treatment responses. The remaining unexplained variance (approximately 82%) likely reflects unmeasured clinical factors, genetic variations, environmental exposures, and stochastic biological processes. Rather than suggesting direct clinical deployment for personalized medicine, our models may serve as complementary tools to assist clinical decision-making by identifying patients at elevated risk, thereby supporting more intensive monitoring and early intervention strategies. However, clinicians should exercise caution when interpreting predictions for patients with extreme values (>1,000 mg/L), where systematic underestimation occurs. In such cases, the model serves as a trigger for enhanced monitoring rather than a basis for autonomous decisions.

## Data Availability

The original contributions presented in the study are included in the article/Supplementary material, further inquiries can be directed to the corresponding author.

## References

[1] World Health Organization. Global Tuberculosis Report 2023. Geneva: WHO (2023).

[2] Wang L Zhang H Ruan Y Chin DP Xia Y Cheng S . Tuberculosis prevalence in China, 1990–2010; a longitudinal analysis of national survey data. Lancet. (2014) 383:2057–64. doi: 10.1016/S0140-6736(13)62639-2 24650955

[3] Günther G Ruswa N Keller PM. Drug-resistant tuberculosis: advances in diagnosis and management. Curr Opin Pulm Med. (2022) 28:211–7. doi: 10.1097/MCP.0000000000000866 35220372 PMC9415219

[4] Obermeyer Z Emanuel EJ. Predicting the future—big data, machine learning, and clinical medicine. N Engl J Med. (2016) 375:1216–9. doi: 10.1056/NEJMp1606181 27682033 PMC5070532

[5] Deo RC. Machine learning in medicine. Circulation. (2015) 132:1920–30. doi: 10.1161/CIRCULATIONAHA.115.001593 26572668 PMC5831252

[6] Rauschert S Raubenheimer K Melton PE Huang RC. Machine learning and clinical epigenetics: a review of challenges for diagnosis and classification. Clin Epigenetics. (2020) 12:51. doi: 10.1186/s13148-020-00842-4 32245523 PMC7118917

[7] Rajpurkar P Chen E Banerjee O Topol EJ. AI in health and medicine. Nat Med. (2022) 28:31–8. doi: 10.1038/s41591-021-01614-0 35058619

[8] Shilo S Rossman H Segal E. Axes of a revolution: challenges and promises of big data in healthcare. Nat Med. (2020) 26:29–38. doi: 10.1038/s41591-019-0727-5 31932803

[9] Qureshi H. AI-driven analysis of buoyancy-convective flow of ternary-hybrid nanofluid in a porous medium over stretching cylinder. Nonlinear Dyn. (2025) 113:28907–24. doi: 10.1007/s11071-025-11620-3

[10] Qureshi H. Artificial neural network simulation modeling of heat transfer effects on a magnetohydrodynamic Casson liquid film flow over a stretching surface. Multiscale Multidiscip Model Exp Des. (2026) 9:49. doi: 10.1007/s41939-025-01110-9

[11] Luo Y Xue Y Song H Tang G Liu W Bai H . Machine learning based on routine laboratory indicators promoting the discrimination between active tuberculosis and latent tuberculosis infection. J Infect. (2022) 84:648–57. doi: 10.1016/j.jinf.2021.12.046 34995637

[12] Kelly CJ Karthikesalingam A Suleyman M Corrado G King D. Key challenges for delivering clinical impact with artificial intelligence. BMC Med. (2019) 17:195. doi: 10.1186/s12916-019-1426-2 31665002 PMC6821018

[13] Khan FU Khan FU Aqeel MT Hayat K Chang J Rehman AU . A randomized controlled trial to evaluate the impact of pharmacist-led clinical interventions on the health-related quality of life among TB patients. Front Pharmacol. (2023) 14:1171985. doi: 10.3389/fphar.2023.1171985 37292150 PMC10246751

[14] Devarbhavi H Aithal G Treeprasertsuk S Takikawa H Mao Y Shasthry SM . Drug-induced liver injury: Asia Pacific Association of Study of Liver consensus guidelines. Hepatol Int. (2021) 15:258–82. doi: 10.1007/s12072-021-10144-3 33641080

[15] Tweed CD Wills GH Crook AM Dawson R Diacon AH Louw CE . Liver toxicity associated with tuberculosis chemotherapy in the REMoxTB study. BMC Med. (2018) 16:46. doi: 10.1186/s12916-018-1033-7 29592805 PMC5875008

[16] Bizuneh FK Masresha SA Yayeh BM Bizuneh TK. Active tuberculosis incidence among treatment failure experienced patients in North Wollow zone: a multicenter historical cohort. Health Sci Rep. (2024) 7:e1997. doi: 10.1002/hsr2.1997 38562614 PMC10982461

[17] Kolloli A Subbian S. Host-directed therapeutic strategies for tuberculosis. Front Med (Lausanne). (2017) 4:171. doi: 10.3389/fmed.2017.00171 29094039 PMC5651239

[18] Gelaw Y Getaneh Z Melku M. Anemia as a risk factor for tuberculosis: a systematic review and meta-analysis. Environ Health Prev Med. (2021) 26:13. doi: 10.1186/s12199-020-00931-z 33485299 PMC7824931

[19] Umemura T Kakizaki H Mutoh Y Mizuno T Ito Y Hioki T . Effectiveness and safety of the simulation-based first-dose design of voriconazole. J Infect Chemother. (2025) 31:102453. doi: 10.1016/j.jiac.2024.06.016 38944383

[20] Swartling M Hamberg AK Furebring M Tängdén T Nielsen EI. Model-informed precision dosing of vancomycin in clinical practice: an intervention development study. Int J Clin Pharm. (2025) 47:178–86. doi: 10.1007/s11096-024-01822-x 39514047 PMC11741990

